# A Review on the Geometry of the Conharmonic Curvature Tensor of Almost Hermitian and Almost Contact Metric Manifolds

**DOI:** 10.1016/j.mex.2026.104078

**Published:** 2026-07-28

**Authors:** Habeeb M. Abood, Farah Hasan AlHusseini

**Affiliations:** a University of Basrah, College of Education for Pure Sciences, Department of Mathematics, Basrah, Iraq; b University of Al-Qadisiyah, College of Education, Department of Mathematics, Al-Qadisiyah, Iraq

**Keywords:** Almost Hermitian manifold, almost contact metric manifold, conharmonic curvature tensor

## Abstract

This literature review focuses on six studies of the conharmonic curvature tensor in almost Hermitian and almost contact metric manifolds. The conharmonic curvature tensor is a more effective constant invariant for detecting conformal curvature events than traditional invariants. The principal methodological concern is the absence of a thorough investigation that integrates previous research outcomes regarding the conharmonic curvature tensor. Conformal flatness and its related invariants impose significant structural constraints on the Vaisman-Gray, nearly Kähler, and locally conformal cosymplectic manifolds, frequently leading to Einstein or Einstein-normalized metrics or a reduction to locally conformal Kähler representations. A nearly conformal Kähler structure with a vanishing conharmonic tensor has been established, and harmonic flattening in dimensions more than four leads to a locally conformal Kähler structure with vanishing scalar curvature in the Vaisman-Gray case. These manifolds are proven to be equivalent to Einstein manifolds under additional assumptions, and necessary and sufficient conditions for the existence of point-homogeneous segmental tensors are specified. Three harmonic constants that determine the various η-Einstein classes of type (α,β) have been established in the normal state of a flat conharmonic tensor, whereas locally conformal manifolds must satisfy harmonic flatness, normality of distribution, and η-Einstein's condition. A classification based on harmonic behavior is constructed in a nearly locally identical space; paracontact criteria are defined, and manifolds are shown to be homogeneous paracontact. Finally, we present the main conclusion derived from the results of this literature review, along with an illustrative application. The findings indicate that conharmonic curvature tensor helps establish novel invariant-based classifications for future studies in differential geometry.

## Graphical abstract

**Figure:**
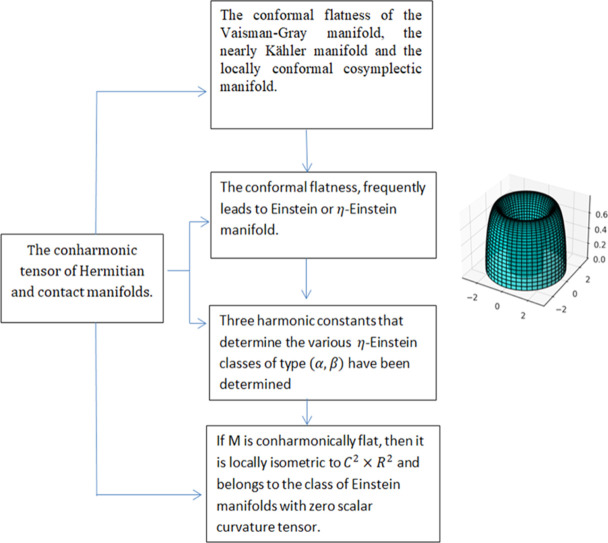

**Specifications table**

**Table:** 

**Subject area**	*Mathematics*
**More specific subject area**	Contact Geometry *and Hermitian Geometry*
**Name and reference of original method**	*The name of the method is:* Adjoined G-structure space*Reference* • Kirichenko V.F., Methods of Generalized Hermitian Geometry in the Theory of Almost-contact Manifold, Journal of Soviet Mathematics, 5, (1988), 1885-1919. • Ishi, T., On conharmonic transformations, Tensor (N.S.), 73080, (1957).
**Resource availability**	*N/A*

## Method details

### Concepts

Recent work has highlighted the conharmonic curvature tensor as a crucial tool in differential geometry, owing to its ability to detect geometric phenomena beyond the scope of standard conformal invariants. While the Weyl conformal tensor indicates how far a manifold is from being conformally flat, the conharmonic tensor is derived from conharmonic maps that keep harmonicity intact, thereby offering a finer analytical vantage point for studying curvature in both complex and contact geometries. Its relevance is underscored by the fact that conharmonic invariants impose remarkably tight constraints on a manifold’s architecture, frequently yielding classification theorems that, when carefully dissected, disclose unexpected symmetries and structural truths [1].

In almost Hermitian geometry, the study of conharmonic recurrence and holomorphic sectional conditions was the first step toward understanding how the conharmonic tensor interacts with known geometric structures. This investigation serves as an initial step toward comprehending the interplay between these elements. The existence of pointwise holomorphic sectional conharmonic tensors imposes necessary and sufficient conditions that restrict the geometry, often forcing it to be Einstein [2,3]. Such results reveal the fact that geometric conditions based on curvature in the conharmonic tensor are not just theoretical constructs, but rather they have important structural implications for every point in the manifold.

This framework was then further extended to Vaisman-Gray manifolds, which are a particularly intriguing subclass of locally conformal Kähler manifolds. Furthermore, in those cases, it was proved that vanishing conharmonic curvature tensor is necessary for scalar flatness and a reduction to the conformal Kähler manifold; this is a way of tying conharmonious tandems together with some of their most essential restrictions of complex geometry [2,4]. These results not only confirmed the effectiveness of the tensor but also indicated that it has a particularly strong influence on the intermediate class of almost Hermitian manifolds, where conformal and contact properties intersect.

Meanwhile, the investigation has been shifted to almost contact metric manifolds, particularly to locally conformal almost cosymplectic manifold (*LCAC*) and special locally conformal almost cosymplectic manifold (*LCACS*). In this paper, we find the strict conditions for conharmonic flatness over these manifolds and some more general conditions of Φ-invariance and quasi-invariance [[5], [6], [7]]. These findings further showed that the presence or absence of conharmonic tensors is directly related to whether or not *η*-Einstein form, or Φ-quasi invariants, can exist, which strengthened contact geometry's classification theory significantly. Furthermore, the explicit construction of invariants and subcategories, such as $CC_{i},\;i=1,2,3,$ confirmed the observation that the tensor not only characterises geometric flatness but also acts as a powerful agent in maintaining structural solidarity. Kirichenko et al. investigated geometric properties of the conharmonic curvature tensor in almost Hermitian manifolds [8]. In contrast, Parkasha and Hadimani examined the conharmonic curvature tensor in a well-known category of almost contact metric manifolds, the Kenmotsu manifold [9]. Siddiqui and Ahsan investigated the conharmonic curvature tensor within the context of four-dimensional spacetime relativity [10]. Abass and Abood studied the generalized curvature tensor of the Kenmotsu-type manifold and found that if it is flat, an *η*-Einstein manifold can be created [11].

Recently, the conharmonic curvature tensor has been studied on different kinds of almost contact metric and almost Hermitian manifolds. Bushba et al. examined the conharmonic tensor on the Sasakian manifold, which is essentially contact metric. In particular, the conharmonic tensor is linked to the semi-symmetric CK-connection in the Sasakian manifold [12]. M. Banaru and G. Banaru investigated the conharmonic curvature tensor of a 6-dimensional plannar Hermitian submanifold of Gayley algebra. They determined the spectrum of the conharmonic curvature tensor for this type of manifold [13].

The present critical review is a combination of six relevant publications to provide a picture of the expanding importance of the conharmonic tensor in different geometric contexts. Chronologically, we observe both the progression of the research and the different ways the results are described, particularly how the investigation of recurrence in the almost Hermitian manifold has been developed to classify *LCAC* and *LCACS*. From this combination, we conclude that the conharmonic curvature tensor is an analytical invariant and a classification tool for the research fields in both almost Hermitian and almost contact geometries.

Many studies have addressed this important type of curvature tensor and focused on its various geometric properties, but they have operated independently, and we have not found a unified study linking the common geometric results of these studies.

Our current study highlights this gap by summarizing and comparing six noteworthy studies, drawing a common geometric conclusion as indicated in the section entitled "Unified Significant Result," and supporting this with an application provided in this study. Specifically, the present study focused on providing a systematic analytical comparison. Unlike previous studies that focused on a single class of manifolds, this study combines six separate analyses into one template, compares the results across different geometric contexts, and establishes a unified classification that is applicable to both almost Hermitian manifolds and almost contact metric manifolds.

### Review methodology

The main objective of this review is to compile and analyse the key findings related to the conharmonic curvature tensor in almost Hermitian and almost contact metric manifolds.

• **Reason for selecting the six studies:** The study focused on selecting six important studies that concentrate on the conharmonic curvature tensor and the most significant findings derived from them. The main reason for this selection is that these studies represent a cohesive group that addressed the geometric properties of the same tensor.
• **Extracting the main content and results:** The key elements identified in each paper are the geometric structure, the hypotheses presented on the manifolds, the basic definitions, the main results, and Identifying the geometric properties of the conharmonic tensor in every analysis.
• **Comparsion and analysis:** The results were compared and analysed according to manifold type, curvature hypotheses, geometric properties of the conharmonic curvature tensor, and results related to Einstein and η -Einstein. This comparison allows for the identification of similarities and differences between these studies.
• **Using an adjoined *G*-structure space method**: This method interpreted the results through a uniform classification framework across the six studies, leading to a unified understanding of the findings. This method helps classify geometric structures using the requirements of the conharmonic operator.
• Conclusions: After determining the primary findings from those investigations, they were combined into a coherent conclusion, stating that the conditions imposed on the conharmonic curvature tensor yeild important geometric properties and classifications in both almost Hermitian and almost contact metric manifolds. This result clarifies the review methodology and improves the study's reproducibility on different curvature tensors.
• **Methodology contribution**: The unique feature of this methodology lies not in formulating novel curvature identities, but in providing a unified analytical strategy within a single geometric context for formerly separate studies. This review systematically correlates the assumptions and geometric characteristics of the analyzed tensor with the conditions related to Einstein-type manifolds derived from established manifolds, thereby creating methodological connections overlooked in previous studies.

## Preliminaries

The contents of this section provide definitions and prerequisites for the present research survey in every aspect of almost Hermitian manifolds, the conharmonic curvature tensor, and almost contact metric manifolds.

In this study, we will refer to Mas the smooth manifold, gas the Riemannian metric, and X, Y, and Z as the smooth vector fields on M.

### Fundamental Definitions

Definition 2.1 [14] An almost Hermitian manifold (AH-manifold) is a triple {M,J,g≤.,.>}, where M is a smooth manifold, J is an almost complex structure satisfying $J^{2}=-id,$ and g≤.,.> is a Riemannian metric on M, such that the subsequent equality is satisfied:

<JX,JY≥〈X,Y〉,∀X,Y∈X(M),

where X(M)is a set of vector fields on the manifold M.

Definition 2.2 [14] A Vaisman-Gray structure is an AH-structure (J,g≤.,.>) such that the following equality holds:

$$\nabla_{X}\left(F\right)\left(X,Y\right)=\frac{-1}{2\left(n-1\right)}\left\{\langle X,Y\rangle \delta F\left(Y\right)-\langle X,Y\rangle \delta F\left(X\right)-\langle JX,Y\rangle \delta F\left(JX\right)\right\}$$

where ∇ *i*s a Riemannian connection of the metric g; that means a unique connection satisfies ∇g=0.,

F(X,Y)≤JX,Y> is a K$\ddot{a}$hler form,δis a coderivative associated with the exterior derivative and used to measure the divergence of differential forms with respect to the Riemannian metric g, andX,Y∈X(M)*.*

Definition 2.3 [14] An AH-structure (J,g≤.,.>) is called a structure of class$\;W_{1}$or a nearly K$\ddot{a}$hler (NK-structure) if the K$\ddot{a}$hler form fulfils the condition

$$\nabla_{X}\left(J\right)=0;\;X\in X\left(M\right).$$

The above equation is equivalent to the condition $\left(\nabla_{X}J\right)\left(Y\right)+\left(\nabla_{X}J\right)\left(X\right)=0$, X,Y∈X(M).

Definition 2.4 [15] An almost contact metric structure (ACM*-*structure) is a quadruple S=(ζ,η,Φ,g) of tensor fields, where ζ is a characteristic vector, η is a covector called a contact one-form, Φ is an endomorphsim of a module X(M) which is a tensor of kind (1,1), and g=〈.,.〉 is a (pseudo) Riemannian metric. Furthermore, the following conditions hold:

1. Φ(ζ)=0;

2. η(ζ)=1;

3. $\Phi^{2}=-id+\eta ⊗\;\zeta ;$

4. η∘Φ=0;

5. g(ΦX,ΦY)=g(X,Y)−η(X)η(Y),forX,Y∈X(M).

An ACM-manifold is a (2n+1)-dimensional manifold M with the quadruple mentioned above.

Definition 2.5 [16] An almost contact metric structure S=(ζ,η,Φ,g) is called an almost cosymplectic structure (*ACs*-structure), if the following conditions hold:

1. dη=0;
2. dΩ=0.

Definition 2.6 [17] An almost contact metric structure is called a normal if$\;N_{\Phi }+2d\eta ⊗\xi =0$, where $N_{\Phi }$ is the Nijenhuis tensor of the operator Φ. It quantifies the impediment to the integrability of the almost contact structure and is defined by the formula

$$N_{\Phi }\left(X,Y\right)=\left[\Phi X,\Phi Y\right]+\Phi^{2}\left[X,Y\right]-\Phi \left[\Phi X,Y\right]-\Phi \left[X,\Phi Y\right],$$

where [X,Y]=X.Y−Y.X denotes the Lie bracket.

Definition 2.7 [18] A normal almost cosymplectic structure is called a cosymplectic structure.

Definition 2.8 [19] A conformal transformation of an *ACM* -structure S=(ζ,η,Φ,g) on a manifold M is the passage from *S* to an *AC*-structure $\tilde{S}=\left(\tilde{\zeta },\;\tilde{\eta },\tilde{\Phi },\;\tilde{g}\right)$ such that $\tilde{\;\eta }=e^{-\sigma }\eta ,\;\tilde{\;\zeta }=e^{\sigma }\zeta ,\;\tilde{\Phi }\;=\;\Phi ,\;\tilde{\;g}=e^{-2\sigma }g,$ where σ is the determining function of the conformal transformation.

Definition 2.9 [19] An *AC*-structureS=(ζ,η,Φ,g)on manifold M is said to be a locally conformal almost cosymplectic (LCAC- structure) if the restriction of this structure to some neighbourhoodU of an arbitrary point p∈M admits a conformal transformation into an almost cosymplectic structure. A manifold equipped with an LCAC- structure is called an LCAC- manifold.

Definition 2.10 [20] A Riemannian curvature tensor R of a smooth manifold M is a 4-covariant tensor $R:T_{p}\left(M\right)\times T_{p}\left(M\right)\times T_{p}\left(M\right)\times T_{p}\left(M\right)\to R$*,* defined as follows:

R(X,Y,W,Z)=g(R(W,Z)Y,X),

where $T_{p}\left(M\right)$ denotes the tangent space of the manifold Mat the point p, X,Y,W,Z∈X(M).

Definition 2.11 [21] A tensor of type (2,0), obtained by contracting the Riemannian curvature tensor as $r_{ij=\;}R_{ijk}^{k}=g^{kl}R_{kijl}$, is known as a Ricci tensor.

Definition 2.12 [1] A conharmonic tensor of type (3,1) on a Riemannian manifold *M* of dimension m is defined as the form:

$$C_{jkl}^{i}=R_{jkl}^{i}+\frac{1}{n-2}\left(\delta_{k}^{i}r_{jl}-\delta_{l}^{i}r_{jk}+g_{jl}r_{k}^{i}-g_{jk}r_{l}^{i}\right),$$

where r,R,and g indicate the Ricci tensor, the Riemannian curvature tensor, and the Riemannian metric, respectively. If the manifold is almost contact metric, then m=2n+1, If the manifold is almost Hermitian, then m=2n.

Definition 2.13 A Riemannian manifold is called a flat conharmonic if the conharmonic tensor is identical to zero.

Definition 2.14 [22] A contact metric manifold is said to be η-Einstein if

r=αg(X,Y)+βη(X)η(Y),

where α and β are smooth functions. If β=0, Mis regarded as an Einstein manifold.

Definition 2.15 [4] A manifold Mpossesses a pointwise holomorphic sectional conharmonic (PHT- tensor) if h does not depend on X, signifying that

$C\left(X,JX,X,JX\right)=h∥X{∥}^{4}$;$\;X\in X\left(M\right),\;h\in C^{\infty }\left(M\right)$.

Definition 2.16 [6] A normal LCAC-manifold (LCACS-manifold) is called a normal LCAC-manifold of class $\;C_{2}$ If its conharmonic curvature tensor satisfies the identity

$C\left(\Phi^{2}X,\Phi^{2}Y\right)\Phi^{2}Z+C\left(\Phi^{2}X,\Phi Y\right)\Phi Z+C\left(\Phi X,\Phi^{2}Y\right)\Phi Z-C\left(\Phi X,\Phi Y\right)\Phi^{2}Z=0,\;\forall X,Y,Z\in X\left(M\right)$.

Definition 2.17 [7] In the adjoined G-structure space, the LCAC-manifold is classified as a manifold of class:

$CC_{1}$ if, $C_{abcd}=C_{\hat{a}bcd}=C_{\hat{a}\hat{b}cd}=0$;

$CC_{2}$ if, $C_{abcd}=C_{\hat{a}bcd}=0$;

$CC_{3}$ if, $C_{\hat{a}bcd}=0$.

For more information about the adjoined G-structure space, refer to reference [23].

Definition 2.18 [7] The conharmonic tensor is called a Φ-quasi invariant if

$\langle C(\Phi X,\Phi Y)\Phi X,\Phi Y\rangle -\langle C\left(\Phi^{2}X,\Phi^{2}Y\right)\Phi^{2}X,\Phi^{2}Y\rangle =\langle C\left(\Phi^{2}X,\Phi Y\right)\Phi^{2}X,\Phi Y\rangle -\langle C\left(\Phi X,\Phi^{2}Y\right)\Phi X,\Phi^{2}Y\rangle =0,$

∀X,Y∈X(M).

Definition 2.19 [7] An LCAC-manifold is known as a conharmoniclly Φ-paracontact if its conharmonic tensor fulfils the condition

$$\langle C\left(\Phi^{2}X,\Phi^{2}Y\right)\Phi^{2}W,Z\rangle =\langle C\left(\Phi^{2}X,\Phi^{2}Y\right)\Phi W,\Phi Z\rangle -\langle C\left(\Phi X,\Phi^{2}Y\right)\Phi W,\Phi^{2}Z\rangle -\langle C\left(\Phi X,\Phi^{2}Y\right)\Phi^{2}W,\Phi Z\rangle =0,$$

∀X,Y,W,Z∈X(M).

### Summary and Comparison of the Findings From the Six Studies

The following table summarizes and compares the most important results from these studies, clarifying the review methodology. The table is divided according to reference numbers [2] through [7], the type of manifold, the main hypothesis, the property of the conharmonic curvature tensor, and the main conclusion.

**Table:** 

Ref. No.	Manifold type	Main hypothesis	conharmonic tensor property	Main conclusion
2	Nearly-Kähler	conharmonic recurence	Recurent tensor	Kähler
3	Vaisman–Gray	Vanishing conharmonic tensor	Flat conharmonic	Scalr flat locally conformal Kähler
4	Vaisman–Gray	Pointwise holomorphic sectional conharmonic tensor	Einstein condition	Einstein manifold
5	Locally conformal almost cosymplectic	Flat conharmonic tensor	η-Eienstien condition	η-Eienstien manifold
6	Normal locally conformal almost cosymplectic	Constant tensor	η-Eienstien condition	Classification of the manifolds
7	Spicial type of locally conformal almost cosymplectic	Quasi-invariant tensor	φ-invariant Ricci tensor	Classification according to Spicial Type of manifold

## Main Results

This section delineates the salient features of six extant investigations concerning the conharmonic curvature tensor in almost Hermitian and almost contact metric manifolds. The results are presented as the theorem and its mathematical framework. Subsequently, we identify our most significant common result.

### Fundamental Results

Theorem 3.1 [2]

The conharmonic nearly Kähler manifold is either a conharmonic symmetric manifold or a conharmonic recurrent Kähler manifold.

This theorem proves that the conharmonic tensor on a special class of almost Hermitian manifolds, i.e., a almost Kähler manifold satisfying a recurrence condition, is not invariant, and the manifold turns out to be a conharmonic symmetric manifold or a conharmonic recurrent Kähler manifold. The preliminary situation shows that the conharmonic tensor is parallel i.e., ∇L=0.

Theorem 3.2 [3]

If a Vaisman–Gray manifold M is conharmonically flat and dim(M)>4, then it is locally conformal Kähler with vanishing scalar curvature (an explicit nearly Kähler example with a vanishing conharmonic tensor is given).

The main conclusion of this theorem is that the flatness condition of the conharmonic curvature tensor determines the geometric properties of curvature and also reduces the category of general manifolds to a more special category, i.e., locally conformal Kähler manifolds of zero scalar curvature. As we have already mentioned, an important property of the conharmonic curvature tensor is that it does not have the scalar curvature part of the Riemannian curvature tensor.

Theorem 3.3 [4]

For a Vaisman–Gray manifold with a pointwise holomorphic sectional conharmonic tensor, the paper provides the necessary and sufficient conditions for such a structure; in particular, under these conditions, the manifold is Einstein.

This theorem produces a notable and robust conclusion in theoretical physics: if a Vaisman-Gray manifold (a specific category of nearly Hermitian manifold) possesses a pointwise holomorphic sectional conharmonic tensor, it qualifies as an Einstein manifold, indicating it adheres to the Einstein equation. Consequently, we deduce that the curvature tensor functions not just as an indicator of curvature, akin to the Riemannian curvature tensor, but also enables us to recognize an essential category of manifolds: Einstein manifolds.

Theorem 3.4 [5]

On an LCAC-manifold, the following are true:

1. The conharmonic tensor is flat if and only if:$A_{bc}^{ad}\;=0,$
$B_{abc}=0,$
$B_{ab}=0,$
$\sigma_{a}=0,$
$\sigma_{00}=\;-\left(n\;+\;\frac{1}{2}\right)\;\sigma_{0}^{2}\;\left(for\dim(M)>\;3\right)$,

where $A_{bc}^{ad}$ denotes the components of the holomorphic sectional tensor, $B_{abc}$ denotes the structure tensor[23].

2. If the *LCAC* –manifold M is Φ-invariant and conharmonically flat (with dim(M)<5), then it is *η*-Einstein of type (α,β) with:

$\alpha =-\frac{2n-1}{2},\;\beta =\frac{\left(n+2)(2n-1\right)}{2n}.$

Theorem 3.4 provides the following information:

- A flat conharmonic tensor establishes both the necessary and sufficient criteria for particular components of certain tensors, which are present in the structural equations of a locally conformal Kähler manifold, to also be flat.

- Should the conharmonic tensor be flat and the manifold exhibit *η*-invariance, it follows that the manifold is classified as *η*-Einstein; this classification underscores that the conharmonic tensor serves not just as a curvature metric but also as a criterion for manifold classification.

Theorem 3.5 [6]

For normal LCAC- manifolds, three conharmonic invariants are distinguished, and three corresponding classes are constructed; every manifold in these classes is *η*-Einstein of type (α, β). The values α and β have been computed for each class. For example, class $C_{3}$ is *η*-Einstein with specific values of α and β.

This theorem leads us to the conclusion that imposing the "normal" condition on a locally conformal Kähler manifold, a particular subset of almost contact metric manifolds, allows the conharmonic tensor to categorize the manifold into three separate classifications. In every category, the manifold is demonstrated to be *η*-Einstein, although each illustrates a specific situation dependent on the parameters essential to the manifold's structure. This validates that applying particular conditions to a certain category of manifold can result in the emergence of alternative types; it also reinforces our previous finding that this tensor functions not only as an instrument for assessing curvature but also as a method for recognizing and delineating further categories of manifolds.

Theorem 3.6 [7]

For *LCAC*-manifolds, the authors classify the geometry by the conharmonic tensor and provide necessary conditions for Φ-quasi invariance in classes $CC_{i},\;i=1,\;2,\;3$. Moreover, every manifold of class $CC_{1}$ is conharmonically Φ-paracontact. In addition, if the manifold is $CC_{3}$ with Φ-invariant Ricci tensor, then it is Φ-quasi invariant.

The main result of this theorem is to give further confirmation of the fact that the conharmonic tensor is not only used to measure the curvature but also to classify the manifolds and to demarcate other spaces under some conditions. As a result of the Φ -quasi-invariance criterion, we classify locally conformal Kähler manifolds into three different classes, and each class has its specific properties. For example, the first class gives rise to a Φ -paracontact manifold, and so on.

### Unified Significant Result

Let (M,g) be an n-dimensional almost Hermitian or almost contact metric manifold with a conharmonic curvature tensor. If, after satisfying either of these conditions, the conharmonic tensor is said to be conharmonically flat and Φ-quasi invariant, then the possible models for M are as follows:

1. M has a flat metric and is locally isometric to $C^{m}\times R^{k}$.
2. M is an η–Einstein manifold of the form r=αg+βη⊗η.
3. M is an Einstein manifold with constant scalar curvature.

In each case, the classification of the manifold in the G-structure space is uniquely determined by the properties of the conharmonic tensor, and these specifications hold for either almost Hermitian or almost contact metric settings.

### Comparative Analysis of Six Studies on the Conharmonic Curvature Tensor

#### Development and Motivation

Rather than considering six distinct lines of inquiry, this review aims to link the studies in a clear, connected way. Theorem 4.1 explains that on a nearly Kähler manifold under certain conditions, the conharmonic tensor is recurrent or symmetric. Theorem 4.2 generalizes the result to Vaisman-Gray manifolds and then gives rise to a locally conformal Kähler manifold with vanishing scalar curvature. Theorem 4.3 concerns pointwise holomorphic curvature and gives conditions for when the examined spaces are a kind of Einstein manifolds. Theorems 4.2-4.6 consider almost contact metric manifolds, focusing on various forms of a normal conformal almost cosymplectic (LCAC and LCACS), all the while emphasizing kinds of separate, distinct distinctions between η-Einstein, Φ-invariant, and conharmonically flat properties. A thorough analysis of these theorems makes it possible to see that there is a logical progression: we started with general recurrence properties, studied curvature properties, which ended in Einstein manifolds, and gave rise to a detailed classification. So this is a first example of passing from the general class to more specific classes of almost contact metric manifolds, namely, classes $C_{i},\;i=1,\;2,\;3.$ This natural progression prompted us to consider the six studies as a whole, and we clarified this implication in our final combined result.

#### Application

This section presents applications of the most significant results from the six studies.

**Fig. 1:**
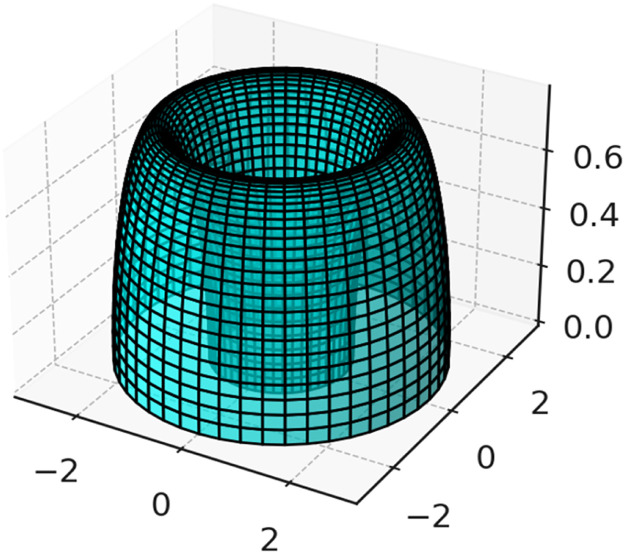
3D representation of the manifold C² × R² with flat conharmonic curvature tensor.

**1- Flat Riemaniian Manifold**: Let $M\;=\;C^{2}\;\times \;R^{2}$ be a 6-dimensional manifold, $C^{2}$ having with the standard Hermitian metric $g_{C}\;$and $R^{2}$ having the Euclidean metric $g_{R}$. Let $g\;=\;g_{C}\;+\;g_{R}$. Define an almost complex structure J on $C^{2}$ by $J\left(\partial /\partial x_{i}\right)\;=\;\partial /\partial y_{i}$ and $J\left(\partial /\partial y_{i}\right)\;=\;-\partial /\partial x_{i}\;$for i=1, 2, and extend trivially to $R^{2}$. The Ricci tensor r vanishes identically; therefore, the Riemannian curvature tensor R is flat, and thus the conharmonic curvature tensor identical to 0. Then, M is conharmonically flat, locally isometric to $C^{2}\;\times \;R^{2}$, and belongs to the class of Einstein manifolds with zero scalar curvture (Fig. 1).

Based on the unified result, we propose that (m,g) denotes an almost Hermitian or almost contact metric manifold characterized by a conharmonic curvature tensor that meets the specified criteria, implying that M has a flat Riemannian curvature tensor, representing a specific instance of flat space. We need to seek the second and third aspects of the consolidated outcome.

**2- Einstein manifold:** Let (M,g) denote an n-dimensional Riemannian manifold, with n>0.

According to the established Einstein field equation, it follows that

$r=-\frac{1}{2}rg+\Lambda g=MT$, where r is the Ricci tensor, g is the Lorentz metric, is the cosmological constant, is the Einstein gravitational constant, and is the energy-momentum tensor. This equation can be used when the manifold is not a Riemannian manifold but a Lorentz manifold. So we can reduce this equation on a Riemannian manifold to the following Einstein equation:

(1) r=λg

where λ is a constant.

Equation (1) can be expressed in component form as follows:

(2) r(X,Y)=λg(X,Y)

where X,Y∈X(M).

By contracting Equation (2) with the metric g, the scalar curvature tensor is expressed as

$$S=g^{ij}\lambda g_{ij}=n\lambda ,$$

where nis the dimension of the manifold M.

According to Equation (2), it follows

$$r=\frac{S}{n}g$$

Using the Ricci operatorR, which is characterized by

g(RX,Y)=r(X,Y),

Consequently, we get

RX=λX.

Geometrically, the aforementioned equation signifies that each tangent direction possesses an identical Ricci curvature tensor.

Consequently, the conharmonic curvature tensor is simplified to the Ricci tensor.

3- η-Einstein manifold

Suppose that $\left(M^{2m+1},\phi ,\xi ,\eta ,g\right)$ is an almost contact metric manifold.

According to Definition 2.14, we have

(3) r=αg+βη×η

In this context, η represents the contact 1-form as described in Definition 2.4, whileα and β are smooth functions.

The contraction of the Ricci tensor r yields the scalar curvature, denoted as

$$K=g^{ij}r_{ij}$$

Based on Equation (3), we can conclude that

$$K=g^{ij}(\alpha g_{ij}+\beta \eta_{i}\eta_{j}$$

Since $g^{ij}g_{ij}=n$, we conclude that

$$g^{ij}\eta_{i}\eta_{j}=1$$

Therefore, we obtain

K=nα+β

The Ricci operator R is given by

g(RX,Y)=r(X,Y)

Thus,

RX=αX+βη(X)ξ

IfX∈Kerη, then it follows that

RX=αX and Rξ=(α+β)ξ

Geometrically, the characteristic vector ξ possesses a Ricci curvature tensor.

If β=0, we only have the Einstein manifold with the equation r=αg.

This explains that the Einstein manifold is a special case of an η-Einstein manifold.

4- Lorentz Spacetime (Einstein manifold)

We can use the same technique to study spacetime relativity in the Lorentz manifold rather than the Riemannian manifolds (almost Hermitian or almost contact metric manifold).

Assume that the conditions on the conharmonic curvature tensor are satisfied, as in the unified result, implying that M is an Einstein manifold. Therefore,

(4) r=λg

where λ is a constant.

Rewrite Equation (4) in the component form; it follows that

(5) r(X,Y)=λg(X,Y)

Contracting Equation (5) by the components metric $g^{ij}$ yields

(6) $$K=g^{ij}\lambda g_{ij}=n\lambda$$

From the theory of general relativity, Einstein’s field equation with cosmological constant is given by

(7) $$r-\frac{1}{2}rg+\Lambda g=MT$$

where T represents the energy momentum tensor, Λ denotes the cosmological constant, $M=\frac{8\pi G}{C^{4}}$ is Einstein’s gravitational constant, and c refers to the speed of light.

Substituting Equations (4) and (6) into (7) gives

$$T=\frac{1}{\kappa }\left(\Lambda -\frac{n-2}{2}\lambda \right)g$$

In 4-dimensional spacetime, we have

$$T=\frac{\Lambda -\lambda }{M}g$$

If the Lorentz manifold represents a vacuum spacetime (T=0), then

$$\lambda =\frac{2\Lambda }{n-2}$$

In 4-dimensional spacetime, we conclude that

λ=Λ

Geometrically, this indicates that the Einstein metric, namely the Lorentzian metric characterized by the conharmonic curvature tensor, is compatible with the vacuum Einstein field equation when a cosmological constant is included.

Consequently, an Einstein metric chosen through a conharmonic curvature constraint is congruent, within the Lorentzian framework, with the vacuum Einstein equation incorporating a cosmological constant.

Now, if Λ=0 and λ=0, it follows that r=0.

Therefore, the Lorentzian manifold is Ricci-flat.

**5- Lorentz Spacetime (** η **-Einstein manifold)**

In accordance with Definition 2.14, an almost contact metric manifold qualifies as an *η*-Einstein manifold if its Ricci tensor satisfies the condition

r=αg+βη⊗η

where α and β are smooth functions.

According to Definition 2.4, we have

η(X)=g(X,ξ),

where ξ is the characteristic vector field.

In a Lorentzian manifold, the energy -momentum tensor of a fluid can be written as follows:

T=(μ+P)η⊗η+Pg,

whereμ is the energy density and P is the pressure.

The expressionsr=αg+βη⊗η and T=Pg+(μ+P)η⊗η have the same basic algebraic decomposition.

The coefficient α controls the Ricci component, while the coefficient β measures the distinguished contribution in the ξ-direction.

## Potential Connections with Mathematical Physics

This study highlights the relationship between mathematical concepts and their applications in mathematical physics, as the study focused on the geometric properties of the conharmonic curvature tensor. As is evident from the mathematical formula of this tensor, it is constructed from the Riemannian curvature tensor and the Ricci tensor. It is also related to the conharmonic transformation, which preserves the harmonicity of functions an plays a major role in classical cosmology, particularly in gravity, thermal conductivity, and fluid mechanics.

Applications of the former are found in theoretical physics, particularly in string theory, while applications of the latter are found in contact Hamiltonian mechanics and energy-dissipating systems.

As the results obtained demonstrate, the conditions imposed on the conharmonic tensor lead to significant geometric rigidity. These results contribute to a broader understanding of geometric structures, benefiting physicists and mathematicians with a shared interest in mathematical physics.

It is important to note that while this study does not investigate direct physical applications, it focuses on the geometric significance of the conharmonic tensor and its prominent role in differential geometry constructs. A pertinent question is where the theoretical physical applications of this study can be observed. As the results indicated, the conditions imposed on the conharmonic tensor lead to Einstein- and *η*-Einstein-type constructs. This is of immense importance in differential geometry, as the Ricci tensor is one of the key quantities appearing in Einstein's equations, well-known in the physics of relativity. Although the geometric structures discussed in this study are not directly related to Einstein's theory of spacetime, the emergence of Einsteinian-type conditions highlights the broad geometric significance of the conharmonic tensor and its connection to curvature theories, which play a fundamental role in modern theoretical physics.

The results of this study indicate that the conditions for conharmonic transformation lead to Einsteinian structures derived from Einstein's equationr=λg. Although this equation does not include the other cosmological constants in Einstein's main equation of spacetime, specifically, the cosmological constant, the gravitational constant, and the speed of light, many of the results have shown Einsteinian-type structures. Since we know that the Ricci tensor is a key component of the conharmonic tensor, these results demonstrate the close relationship between the conharmonic tensor and important geometric structures related to the mathematical formulations of the theory of gravity.

## Discussion of Broader Theoretical Implications

This study could be expanded in the future to include other popular manifolds, such as Kemotsu, Sasakian, and quasi-Sasakian manifolds, which are also important types of almost contact metric manifolds. The results of the review study could also be applied to other curvature tensors, specifically the quasi-conformal, pseudo-quasi-conformal, generalized curvature, and Bochner tensors. The paper provides a solid foundation for investigating the geometric properties of these tensors over a wide range of almost contact metric manifolds. One could apply the review's main conclusion, particularly the theorem that unifies previous discoveries into a single, unified result. The applications could also be used to expand this research to include spaces that satisfy the Einstein field equations in general relativity, specifically Lorentz spaces.

## Limitations of the Study

This review is devoted to six papers on the conharmonic curvature tensor for two types of manifolds: almost Hermitian manifolds and almost contact metric manifolds. The reason for choosing this tensor instead of other curvature tensors is due to the different geometric properties of this tensor; in particular, the curvature is invariant under conharmonic transformations. It is also similar to the Weyl curvature tensor, having similar harmonic and conformal properties. The tensor was introduced by Ishii to address limitations of the Riemannian curvature tensor, which had previously been considered the standard in these investigations, as it fails to preserve curvature under conharmonic transformations. This tensor, as shown by its defining equation, is a function of only the Riemannian curvature tensor R and the Ricci tensorr and not of the scalar curvature tensor, which appears in other curvature tensors. Furthermore, there is another geometrical property: the case of flat space, i.e., with constant curvature, is close to the geometry of spaces of constant curvature. This gives rise to important classes of manifolds, for example, Einstein and η-Einstein manifolds, which have been studied in detail. These manifolds play a crucial role, find applications in the context of spacetime relativity, and relate to Einstein's equation for energy conservation.

## Conclusions

The collection of all six studies shows that the conharmonic curvature tensor makes a considerable change in the geometry of almost-Hermitian and almost-contact metric manifolds. We have found conditions on the recurrence and the holomorphic sectional curvature that distinguish whether the conharmonically flat manifolds have strong structural restrictions. In this article, conharmonically flat almost cosymplectic and locally conformal almost cosymplectic manifolds are systematically classified into some classes of geometry on the basis of previous research. These invariant forms, *η*-Einstein and Φ-quasi conditions among them, have a constricting nature; they are geometric properties preserved under transformations between structures, including the preservation of harmonicity. In particular, flatness and recurrence restrict the allowable configurations to Einstein or *η*-Einstein manifolds, a theoretical realization of Einstein's equation, narrowing the possible geometries to smaller, more tractable classes rather than fully general ones. The common feature of all six results is that the cancellation or restriction of the conharmonic tensor terms always makes the manifolds have such a rigid structure. This supports the conharmonic curvature tensor as a significant classification tool for some types of Riemannian manifolds in differential geometry.

## Ethics authors statements

*The platform's data redistribution policies were followed.*

## Funding statement

This study was conducted without external financial support.

## Data availability

The research outlined in the article did not utilize any data.

## CRediT authorship contribution statement

Habeeb M. Abood and Farah Hasan AlHusseini: Conceptualization, Methodology, Writing – original draft. Habeeb M. Abood and Farah Hasan AlHusseini, Visualization, Habeeb M. Abood and Farah Hasan AlHusseini: Investigation, Software, Habeeb M. Abood and Farah Hasan AlHusseini: Writing – original draft.

## Supplementary material *and/or* additional information [OPTIONAL]

Not applicable

## Declaration of competing interest

The authors assert that they possess no recognized financial conflicts or personal affiliations that may have seemingly impacted the work presented in this study.
